# Evaluating Alternative Registration Planes in Imageless, Computer-Assisted Navigation Systems for Direct Anterior Total Hip Arthroplasty

**DOI:** 10.3390/s24217092

**Published:** 2024-11-04

**Authors:** John E. Farey, Yuan Chai, Joshua Xu, Vincent Maes, Ameneh Sadeghpour, Neri A. Baker, Jonathan M. Vigdorchik, William L. Walter

**Affiliations:** 1 Sydney Musculoskeletal Health, Kolling Institute, Northern Clinical School, Faculty of Medicine and Health, University of Sydney, Camperdown, NSW 2064, Australiabill.walter@hipknee.com.au (W.L.W.); 2 Institute of Future Health, South China University of Technology, Guangzhou 511442, China; 3 Department of Orthopedics and Traumatic Surgery, Royal North Shore Hospital, St Leonards, NSW 2065, Australia; 4 Department of Orthopedic Surgery, University Hospitals Leuven, Herestraat 49, 3000 Leuven, Belgium; 5 Innovation Department, Navbit, Sydney, NSW 2000, Australianeri.baker@navbit.com (N.A.B.); 6 Adult Reconstruction and Joint Replacement Service, Hospital for Special Surgery, New York, NY 10021, USA; vigdorchikj@hss.edu

**Keywords:** computer assisted surgery, imageless navigation, optical sensor, total hip arthroplasty, acetabular orientation, registration plane, implant accuracy

## Abstract

(1) Background: Imageless computer navigation systems have the potential to improve the accuracy of acetabular cup position in total hip arthroplasty (THA). Popular imageless navigation methods include locating the patient in a three-dimensional space (registration method) while using a baseline to angle the acetabular cup (reference plane). This study aims to compare the accuracy of different methods for determining postoperative acetabular cup positioning in THA via the direct anterior approach. (2) Methods: Fifty-one participants were recruited. Optical and inertial sensor imageless navigation systems were used simultaneously with three combinations of registration methods and reference planes: the anterior pelvic plane (APP), the anterior superior iliac spine (ASIS) and the table tilt (TT) method. Postoperative acetabular cup position, inclination, and anteversion were assessed using CT scans. (3) Results: For inclination, the mean absolute error (MAE) was lower using the TT method (2.4° ± 1.7°) compared to the ASIS (2.8° ± 1.7°, *p* = 0.17) and APP method (3.7° ± 2.1°, *p* < 0.001). For anteversion, the MAE was significantly lower for the TT method (2.4° ± 1.8°) in contrast to the ASIS (3.9° ± 3.2°, *p* = 0.005) and APP method (9.1° ± 6.2°, *p* < 0.001). (4) Conclusion: A functional reference plane is superior to an anatomic reference plane to accurately measure intraoperative acetabular cup inclination and anteversion in THA using inertial imageless navigation systems.

## 1. Introduction

Accurate patient-specific acetabular component positioning is essential to the long-term performance of total hip arthroplasty (THA). Malposition of the acetabular component is associated with an increased risk of dislocation [1,2], revision surgery [3], early readmission [4], metallosis [5], and accelerated bearing wear [6]. Clinical studies of computer-assisted navigation have demonstrated improved reproducibility of cup placement within predefined target zones over manual acetabular cup positioning by the surgeon according to variable intraoperative landmarks [7,8]. Large observational cohort studies have reported a lower incidence of dislocation and acetabular revision for primary elective THA performed with computer-assisted navigation [9,10].

Despite these reported benefits, navigation is used in less than 5% of THA procedures due to concerns regarding increased costs, operative time, accuracy, and radiation exposure in image-based systems [11]. Navigation for placement of the acetabular cup via the direct anterior approach (DAA) has traditionally been image-based with the use of intraoperative fluoroscopy [12]. Imageless navigation systems were designed to overcome the limitations of image-based systems by being simple to use, involving no radiation, and ensuring maintenance of sterility. Popular imageless navigation methods include locating the patient in a three-dimensional space (registration method) while using a baseline to angle the acetabular cup (reference plane).

To date, imageless registration can be conducted either via optical or inertial sensor methods. In contrast to optical sensors, inertial sensors are more suited to acquiring a functional reference plane. Compared to the anatomic reference plane that uses anatomical landmarks to define the anterior pelvic plane (APP), the functional reference plane is based on the patient’s position on the table during registration. This functional plane can accommodate pelvic tilt better and, consequently, results in a lower measurement error for two reasons. Firstly, the anatomic reference plane method assumes a neutral (0°) pelvic tilt. However, this assumption may not reflect true intraoperative circumstances in many patients undergoing THA, with pelvic tilt reported as varying from −20.5 degrees to 24.5 degrees in the supine position [13,14]. Moreover, the supine functional reference plane generally correlates well with the standing functional reference plane, as there is a mean posterior rotation of only 5.5 degrees from supine to standing [14,15,16]. Secondly, measurement errors may occur due to variability of patient anatomy and body habitus, especially in obese patients [17,18]. Further, imperfect positioning of the patient on the table can also further compound errors in the functional reference plane.

In this study, we compared the accuracy of three methods (combinations of registration method and reference plane) for determining postoperative acetabular cup positioning in THA via the DAA by concurrently and independently using an imageless optical system and an imageless inertial sensor system.

## 2. Materials and Methods

### 2.1. Study Design

Fifty-one consecutive patients who underwent a primary THA via the DAA for osteoarthritis were prospectively included. Surgery was performed between 1 January 2021 and 30 November 2021. All procedures were performed by a single, experienced surgeon with a high annual volume of hip arthroplasty procedures (WLW) using the DAA in the supine position. The acetabular cup was standardized for all patients (DePuy Synthes Pinnacle, Warsaw, IN, USA). Both an optical device (Stryker OrthoMap, Kalamazoo, MI, USA) and inertial sensor-based device (Navbit, St Leonards, Australia) were used concurrently and independently. The acetabular cup was positioned according to the inertial sensor-based device measurements. Low-dose computed tomography (CT) scans were routinely performed postoperatively at our institution to measure component position, in particular, inclination and anteversion. These measurements were performed by two observers, blinded from the intraoperative measurements. All intraoperative device measurements were compared with postoperative CT measurements as a gold standard. Exclusion criteria included severe spinal deformity (scoliosis, kyphosis, or lordosis) that would alter the pelvic positioning between standing and supine, atypical pelvic anatomy due to prior surgery or deformity, any intra-abdominal hernias adjacent to or overlying the iliac crests, and any contraindication to total hip arthroplasty. The patient data were collected from a research database that was approved by the St Vincent’s Hospital Human Research Ethics Committee (2019/ETH09656). Each patient signed a consent form to use their data for research purposes.

### 2.2. Registration Methods

**Figure 1:**
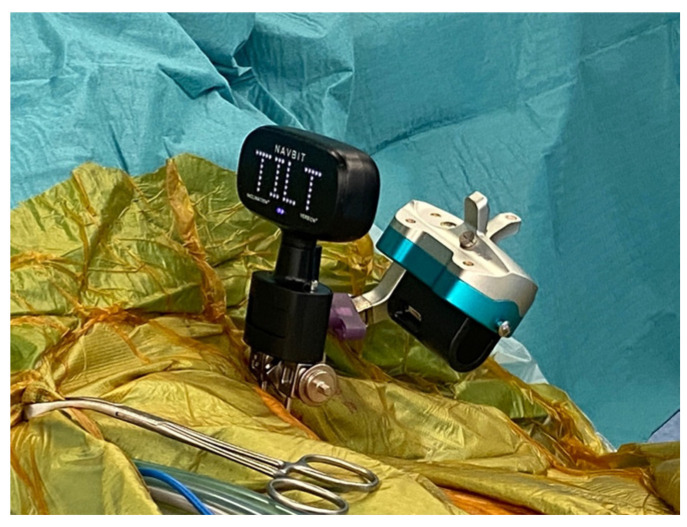
Attachment of the Stryker OrthoMap and Navbit sensor units to the patient’s contralateral iliac crest during supine total hip arthroplasty.

Intraoperatively, two threaded pins were placed in the contralateral iliac crest to facilitate sequential registration of patient position using an optical system and an inertial sensor-based system coupled to the patient (Figure 1). A detailed description of the devices has been provided in previous studies [19,20]. 

An anatomic landmark registration method was used for registration of the optical navigation system. Patient location was identified by palpating the bilateral ASIS and pubic symphysis to acquire for the anterior pelvic plane (APP). To determine the ASIS functional plane, the bilateral ASIS was identified and the table was then raised and lowered so that the optical tracking system could determine the table plane which is perpendicular to the table up–down axis.

**Figure 2:**
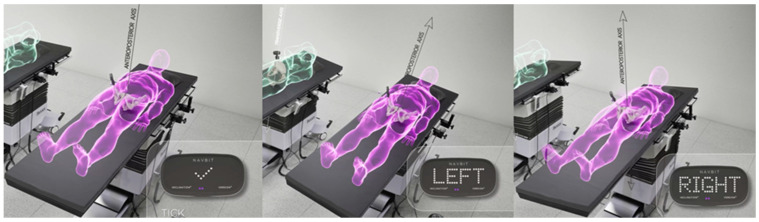
The table-tilt method (TT) of registration. The inertial sensor unit registers the gravity vector with the operation table in the neutral position, parallel to the floor. The operation table is then rotated 10° left and 10° right to generate the table roll axis, parallel to the longitudinal axis of the patient.

For the inertial sensor-based device, a table tilt registration method (TT) was used. The patient was aligned with the operating table and the position was then registered as a 3-dimensional functional pelvic co-ordinate system with three axes. The anteroposterior axis was defined by the gravity vector, the longitudinal axis was generated by tilting the table 10° on each side and measuring the rotation axis of the operating table, and, finally, the transverse axis was calculated by taking the cross-product of the first two axes (Figure 2). 

### 2.3. Reference Planes

**Figure 3:**
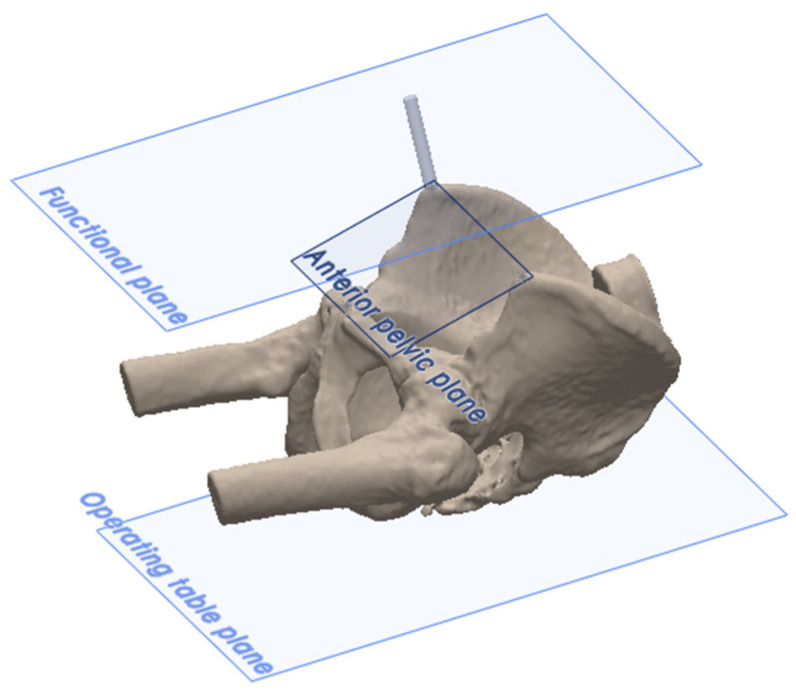
The reference planes, where the two functional pelvic planes (TT functional plane and ASIS functional plane) are parallel to the operating table, with the ASIS functional plane intersecting the ASISs. The anatomic anterior pelvic reference plane (APP) is defined by the ASISs and pubic symphysis.

The anatomic reference plane was the anterior pelvic plane (APP), located by palpating the ASISs and the pubic symphysis (Figure 3). There were two functional reference planes: the ASIS functional plane, which is parallel to the line between the ASISs and parallel to the table plane, and the TT functional plane, which is perpendicular to gravity and aligned with the longitudinal axis of the patient (Figure 3). Following definitive cup impaction, the final acetabular cup position was measured using all three reference planes according to Murray’s definitions of radiographic inclination (RI) and radiographic anteversion (RA) [21]. 

### 2.4. Measurement Methods

The present study compares the intraoperative RI and RA obtained by three measurement methods. Each measurement method was a combination of a registration method and a reference plane: the APP method (anatomic, landmark registration method, and APP reference plane), the ASIS method (anatomic, landmark registration method, and ASIS functional plane), and the TT method (table tilt registration method and TT functional plane).

### 2.5. Radiographic and Statistical Analysis

Intraoperative measurements of RI and RA using the three reference planes were compared to postoperative computed tomography (CT) scans by two authors (DX and YC). For the CT scan measurements, operative anteversion (OA) was converted to RA according to Murray’s formula [21]. The absolute error for each measurement was the difference in absolute values between the intraoperative measurements and the postoperative CT scans. Two repeated-measures analyses of variance (ANOVA) were used to compare differences in the mean absolute errors: one for inclination and one for anteversion. The Greenhouse–Geisser correction for sphericity was used. For significant ANOVA findings, post hoc comparisons were calculated using Tukey’s Honestly Significant Difference (HSD) test. Bland–Altman analysis was used to assess agreement between intraoperative device measurements and postoperative CT values. The clinically relevant limit of accuracy for inclination and anteversion was set at ±10° based on Lewinnek’s analysis [22]. An alpha value of 0.05 was used for all statistical comparisons. Analyses were conducted using Jamovi (Version 1.6.23) and plots were generated using R (Version 4.1.2).

## 3. Results

Fifty-one patients underwent supine THA via the DAA. The mean age of patients was 70.0 years (standard deviation (SD) ± 10.3 years) and the mean BMI was 25.8 (SD ± 4.1). The majority of patients were female (31/51, 61%) and a higher proportion of surgeries were right-sided (31/51, 61%).

**Figure 4:**
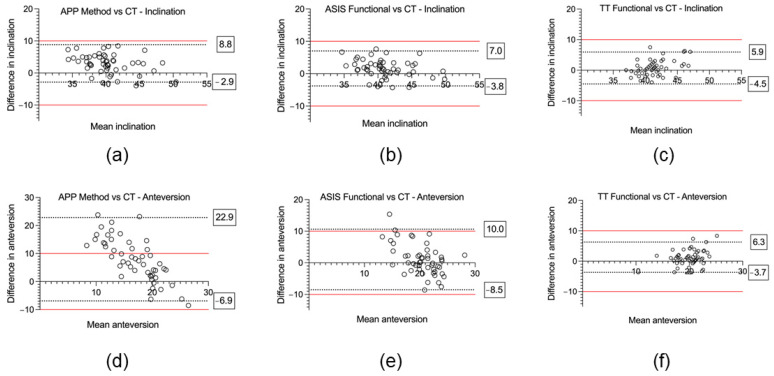
Accuracy of three different reference planes for acetabular cup inclination and anteversion compared to the postoperative computed tomography (CT) reference standard using Bland–Altman plots. (**a**) APP method vs. CT—inclination. (**b**) ASIS method vs. CT—inclination. (**c**) TT method vs. CT—inclination. (**d**) APP method vs. CT—anteversion. (**e**) ASIS method vs. CT—anteversion. (**f**) TT method vs. CT—anteversion. Boxed values represent the 95% limits of agreement, and the red line denotes the ±10° clinically relevant limits of accuracy.

**Figure 5:**
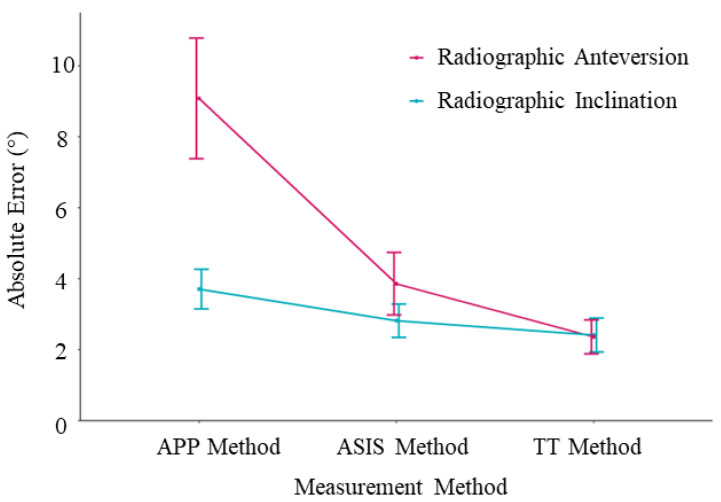
Mean absolute error plots. The error bars represent the 95% confidence intervals.

**Figure 6:**
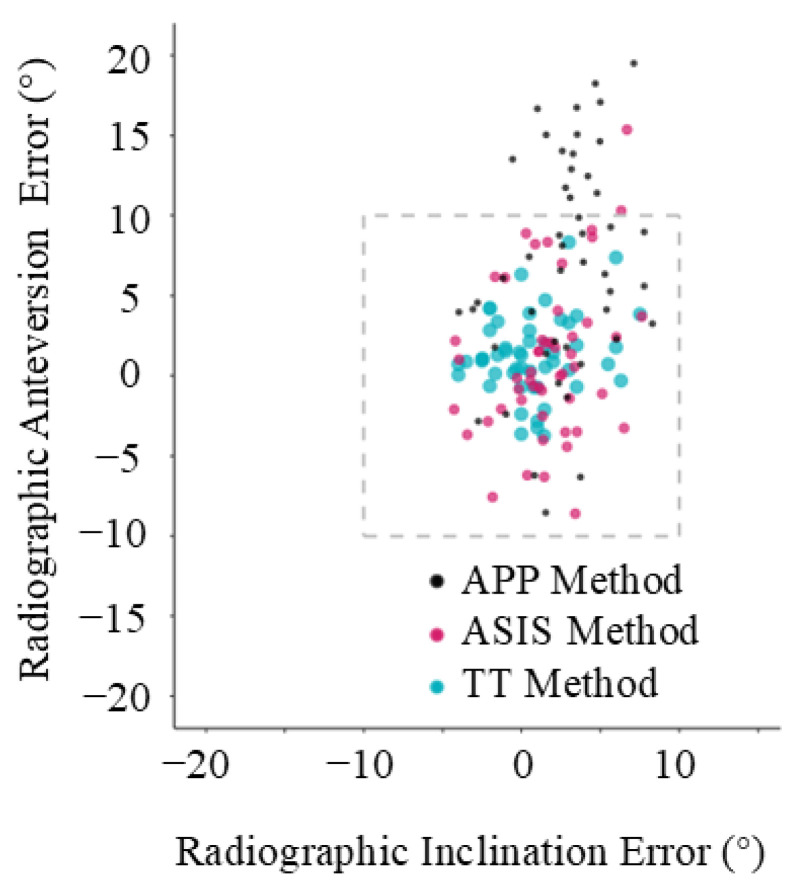
Individual error plot. Each data point represents a patient and shows their error in inclination and anteversion, compared with computed tomography (CT) scan.

Based on CT measurements, the mean radiographic inclination was 41.5° (SD ± 3.2°, range 14.6°) and the mean radiographic anteversion was 19.7° (SD ± 3.3°, range 13.9°). For all three registration planes, mean intraoperative inclination and anteversion, as well as mean absolute error compared to the postoperative CT measurements, are provided in Table 1. The Bland–Altman analysis, the bias, standard deviation, and 95% limits of agreement are also reported in Table 1, and Bland–Altman plots are provided in Figure 4. The mean absolute errors with their 95% confidence intervals are shown in Figure 5, and the errors for each patient are plotted in Figure 6.

For inclination, the mean absolute error was significantly lower using the ASIS method (2.8° ± 1.7°) compared to the APP method (3.7° ± 2.1°); *p* < 0.001. The mean absolute error was significantly lower for the TT method (1.8° ± 1.7°) in contrast to the APP method; *p* < 0.001. There was no significant difference of the mean absolute error between the TT method (2.4° ± 1.7°) and the ASIS method (2.8 ± 1.7°, *p* = 0.17). For the Bland–Altman analysis, the APP method, which uses an anatomic reference plane, demonstrated the highest bias (mean difference (MD) 3.0° ± 3.0°) and widest range for the 95% limits of agreement (−2.9° to 8.8°) (Figure 4a). For the functional planes, the Bland–Altman analysis demonstrated no significant proportional bias for either the ASIS (MD 1.7° ± 3.4°) or TT (MD 1.6° ± 2.8°) methods (Figure 4b,c). No measurements were outside the ±10° clinically relevant limits of accuracy for inclination using either the anatomic or functional reference planes.

For anteversion, the mean absolute error was significantly lower for the ASIS method (3.9° ± 3.2°) compared to the APP method (9.1° ± 6.2°, *p* < 0.001). The mean absolute error was significantly lower for the TT method (2.4° ± 1.8°) as opposed to both the ASIS method (3.9 ± 3.2°, *p* = 0.005) and the APP method (9.1° ± 6.2°, *p* < 0.001). For the Bland–Altman analysis, the APP method demonstrated significant bias (MD 8.0° ± 7.6°) and 95% limits of agreement that exceeded the ±10° clinically relevant limits of accuracy (−6.9 to 22.8°) (Figure 4d). For the functional reference planes, the Bland–Altman analysis demonstrated no significant proportional bias for both the ASIS (1.0° ± 4.9°) and TT methods (1.3° ± 2.5°). The 95% limits of agreement exceeded the ±10° clinically relevant limits of accuracy for the ASIS method (−8.5 to 10.0°) but not for the TT method (−3.7 to 6.3°) (Figure 4e,f). In total, 31% (16/51) of the measurements of anteversion were outside the ±10° clinically relevant limits of accuracy for the APP method, 4% (2/51) for the ASIS method, and 0% (0/51) were outside the limits for the TT method.

## 4. Discussion

A functional reference plane is superior to an anatomic reference plane to accurately measure intraoperative acetabular cup inclination and anteversion in THA via the DAA where no correction is made for sagittal tilt of the pelvis. If the pelvic sagittal tilt is known, then the target can be adjusted and the anatomic reference plane will be more accurate. As shown in Figure 5 and Figure 6, the error in anteversion frequently exceeded 10° using an anatomic reference plane. Despite easier access to the relevant anatomical landmarks in the supine position, close to one third of cup anteversion measurements using the APP method were outside the ±10° recommended by Lewinnek [22] and the widest 95% limits of agreement range was observed (−6.9° to 22.9°). This can be partially attributed to the fact that an anatomic reference plane method assumes a neutral (0°) supine pelvic tilt [23]. As previously mentioned, this does not resemble the reality of a mean supine pelvic tilt of around 4.2 degrees with large intervariability [14]. Additionally, this already variable supine pelvic tilt will have a mean posterior rotation of 5.5 degrees from supine to standing. Although only 5% of patients rotate their pelvis posteriorly more than 13 degrees between the supine and standing position [14], a 13° change in pelvic tilt would correspond with a 10° change in acetabular anteversion, which is clinically significant [24]. This reduced accuracy, when using an anatomic reference plane, is consistent with the theoretical advantage of functional planes in accommodating for patient pelvic tilt [11,25]. Because targets for acetabular cup position typically have a window of ±10°, this error using an anatomic reference plane is likely to be clinically significant [24]. Therefore, the results of our study confirm the theoretical advantage of a functional reference plane for achieving a desired acetabular component positioning intraoperatively, independent of patient pelvic tilt. Alternatively, the pelvic tilt can be measured and the target adjusted, whereby the anatomic method may be used accurately.

Furthermore, our results suggest that the acetabular cup inclination and anteversion were more accurately measured using the TT method rather than using the ASIS method. These results suggest that errors associated with the table tilt registration method, such as patient alignment, have less impact on the accuracy in final cup positioning than errors associated with palpating the ASIS, with a larger differential effect on anteversion compared to inclination. While ±10° tolerance has historically been the reference standard for cup positioning used in the literature based on Lewinnek’s study [22], more sophisticated contemporary studies of the relationship between pelvic positioning during daily activities and the functional orientation of the acetabulum suggest that <5° may be a preferred limit if dislocation and edge-loading are to be prevented in at-risk patients [14]. As both the mean absolute error and mean difference (bias) were within this threshold for both functional methods, the increased accuracy may have clinical significance. Removing the need to palpate anatomical landmarks also reduces the potential to compromise sterility during registration [11,25].

Accuracy using the TT method was comparable to the accuracy observed in previous studies of imageless navigation using functional reference planes without the use of anatomical landmarks [26,27]. In most prior cohort studies, patient registration has most commonly been performed in the lateral decubitus position [26,27]. Clinical studies investigating functional registration planes without the use of anatomical landmarks in the supine position are limited to ones involving an accelerometer-based system [28,29,30] and a 3D mini-optical system [31]. Of these four studies, two were performed via the anterolateral approach [28,30] and two used the DAA [29,31]. The comparative reference standard varies among the studies, being postoperative CT for the accelerometer-based system and postoperative standing AP pelvic radiographs for the optical-based system. Okamoto et al. [29] performing supine THA via the DAA using the accelerometer-based system demonstrated similar mean absolute error for inclination (3.1° ± 2.2°) and anteversion (2.8° ± 2.3°) to our study. Bradley et al. [31] reported lower accuracy using an optical system, with 93% being within 10° of the intraoperative measurement and 85.5% being within 5°. However, Bradley et al. [31] did not account for the effect of changes in pelvic tilt between an intraoperative supine functional plane and a postoperative standing AP radiograph. The resulting bias may explain why they failed to replicate the results of the preclinical simulation study [32]. However, in our study, both functional methods had mean absolute errors that show comparable accuracy to the previous studies.

The strengths of this study include repeated simultaneous reference plane measurement by a high-volume surgeon and assessment of acetabular component positioning by postoperative CT scans performed in the same supine position as the surgery. The limitations of this study include the use of the supine patient position and DAA only, the lack of a control group involving manual cup placement for comparison, limited external validity due to the single surgeon series, absent follow-up of clinically important outcomes, such as dislocation and acetabular revision, and the exclusion of patients with extreme spinopelvic anatomy. Finally, the average BMI of patients in this series is only slightly higher than the healthy weight category (BMI < 25.0). Given that registration of the APP and ASIS method require the palpation of bony landmarks, this study may overestimate the accuracy of these methods due to the lower body habitus of the included patients. Body habitus may affect accuracy of the TT method if significant abdominal girth places pressure on the iliac crest pins after registration, thus altering measurement of the gravity vector. Further studies should be directed towards correlating use of functional registration planes with the prevention of clinically important outcomes such as dislocation and acetabular revision.

## 5. Conclusions

Use of an imageless navigation system, based on a functional registration plane independent of anatomic landmarks, provided the most consistently accurate guide for placement of the acetabular cup in the desired position during supine THA. The use of an anatomic reference plane based on palpable landmarks, without correction for sagittal pelvic rotation, leads to an increase in measurement error that is likely to be clinically significant, with nearly one third of anteversion results outside of a ±10° target window. Future studies should examine the effect of alternative acetabular cup navigation techniques on the incidence of common clinically relevant outcomes, such as dislocation incidence.

## Figures and Tables

**Table 1:** Comparison of acetabular cup angles measured using three registration planes for supine total hip arthroplasty.

Component Position	Post-Operative CT (°)	Reference Plane	Device (°)	Mean Absolute Error ± SD	Bias ± SD (Bland–Altman)	95% Limits of Agreement (Bland–Altman)	*p*-Value (Tukey’s HSD)
Inclination	41.5 ± 3.2	ASIS Anatomic	38.9 ± 3.7	3.7 ± 2.1	3.0 ± 3.0	−2.9 to 8.8	-
		ASIS Functional	39.9 ± 4.0	2.8 ± 1.7	1.7 ± 3.4	−4.5 to 5.9	<0.001 ^a^
		TT Functional	41.1 ± 2.0	2.4 ± 1.7	1.6 ± 2.8	−3.8 to 7.0	<0.001 ^b^
Anteversion	19.7 ± 3.3	ASIS Anatomic	13.0 ± 7.3	9.1 ± 6.2	8.0 ± 7.6	−6.9 to 22.9	-
		ASIS Functional	19.7 ± 4.8	3.9 ± 3.2	1.0 ± 4.9	−8.5 to 10.0	<0.001 ^1^
		TT Functional	19.5 ± 2.4	2.4 ± 1.8	1.3 ± 2.5	−3.7 to 6.3	<0.001 ^2^ 0.005 ^3^

^1^ Tukey’s HSD comparison ASIS functional method versus ASIS anatomic method; ^2^ Tukey’s HSD comparison TT functional method versus ASIS anatomic method; ^3^ TT functional method vs. ASIS functional method.

## Data Availability

Research data are available upon reasonable request by contacting the corresponding author. Data usage must comply with our ethics approval and may be subject to additional approval by our Institutional Review Board.
